# 

**Published:** 1917-02-24

**Authors:** 


### Matrons' and Sisters' Appointments.

Duke of Connaught's Auxiliary Hospital, Bray, Co. Wicklow.—Miss J. Dowley has been appointed matron. She received her training at Guy's Hospital, where she later became staff nurse, and has since been sister at the 4th London General Hospital, Denmark Hill.

Chelsea Hospital for Women.—Miss Ellen West has been appointed matron. She was trained at Wandsworth Union Infirmary and at the Chelsea Hospital for Women, where she has since been staff nurse, ward sister, temporary night sister, temporary assistant matron, and acting matron. Miss West has also held the post of theatre sister at St. James's Infirmary, Balham, and has done private nursing in the United States.

**NOTE.—Particulars of Appointments for insertion in this column will be welcomed.**

### Medical Appointments.

St. Thomas's Hospital House Appointments.—The following have been selected as house officers from Tuesday, February 13, 1917:—*Casualty Officers and Resident Anæsthetists:* H. J. Blampied, M.R.C.S., L.R.C.P.; D. G. Churcher, M.R.C.S., L.R.C.P.; E. S. Orme, B.A. Cantab., M.R.C.S., L.R.C.P.; S. A. T. Ware, M.R.C.S., L.R.C.P. *Resident House Physicians:* W. T. Beswick, B.A. Cantab.; A. Mavrogordato, M.A. Oxon., M.R.C.S., L.R.C.P.; M. W. H. Miles, M.R.C.S., L.R.C.P.; W. G. Woolrich, B.A. Cantab. *Resident House Surgeons:* L. C. Moore, H. C. Jennings, M.R.C.S., L.R.C.P.; G. W. J. Bousfield, P. Sai, M.R.C.S., L.R.C.P. *House Surgeon to Block 8:* D. C. Bluett, M.R.C.S., L.R.C.P. *Obstetric House Physician:* J. R. Harris, M.R.C.S., L.R.C.P. *Ophthalmic House Surgeon:* W. Marriott. *Clinical Assistants:* (Throat), P. G. S. Davis; (Urological Department), Y. J. Cieh, M.R.C.S., L.R.C.P.

### Annual Meeting.

Queen Charlotte's Lying-in Hospital, Marylebone Road, N.W.—Tuesday, February 27, at 3 p.m. precisely, at the hospital.

RATES OF SUBSCRIPTION: Twelve months, 6/6; Six months, 4/-; Three months, 2/-; post free to any address in the United Kingdom. Abroad, Twelve months, 8/8; Six months, 5/-; Three months, 2/6, post free. THE HOSPITAL "Index" to Volume LX., April to September 1916, is now ready, price 2½d. per copy, post free. Address The Manager, "THE HOSPITAL," The Hospital Building, 28/29 Southampton Street, Strand, London, W.C.

